# Systematic review and meta-analysis of outcome-relevant anemia in patients with subarachnoid hemorrhage

**DOI:** 10.1038/s41598-022-24591-x

**Published:** 2022-12-01

**Authors:** Maryam Said, Meltem Gümüs, Jan Rodemerk, Laurèl Rauschenbach, Mehdi Chihi, Thiemo Florin Dinger, Marvin Darkwah Oppong, Börge Schmidt, Yahya Ahmadipour, Philipp Dammann, Karsten Henning Wrede, Ulrich Sure, Ramazan Jabbarli

**Affiliations:** 1 Department of Neurosurgery and Spine Surgery, University Hospital of Essen, 45147 Essen, Germany; 2 Center for Translational Neuro- & Behavioral Sciences (C-TNBS), University Duisburg Essen, Essen, Germany; 3 Institute for Medical Informatics, Biometry and Epidemiology, University Hospital of Essen, 45147 Essen, Germany

**Keywords:** Health care, Medical research, Neurology, Risk factors

## Abstract

Anemia is a common, treatable condition in patients with aneurysmal subarachnoid hemorrhage (SAH) and has been associated with poor outcome. As there are still no guidelines for anemia management after aneurysm rupture, we aimed to identify outcome-relevant severity of anemia in SAH. We systematically searched PubMed, Embase, Scopus, Web of Science, and Cochrane Library for publications before Oct 23rd, 2022, reporting on anemia in SAH patients. The presence and severity of anemia were assessed according to the reported hemoglobin values and/or institutional thresholds for red blood cells transfusion (RBCT). Out of 1863 original records, 40 full-text articles with a total of 14,701 patients treated between 1996 and 2020 were included in the final analysis (mean 445.48 patients per study). A substantial portion of patients developed anemia during SAH (mean pooled prevalence 40.76%, range 28.3–82.6%). RBCT was administered in a third of the cases (mean 32.07%, range 7.8–88.6%), with institutional threshold varying from 7.00 to 10.00 g/dL (mean 8.5 g/dL). Anemia at the onset of SAH showed no impact on SAH outcome. In contrast, even slight anemia (nadir hemoglobin < 11.0–11.5 g/dL) occurring during SAH was associated with the risk of cerebral infarction and poor outcome at discharge and follow-up. The strongest association with SAH outcome was observed for nadir hemoglobin values ranging between 9.0 and 10.0 g/dL. The effect of anemia on SAH mortality was marginal. The development of anemia during SAH is associated with the risk of cerebral infarction and poor outcome at discharge and follow-up. Outcome-relevant severity of post-SAH anemia begins at hemoglobin levels clearly above the thresholds commonly set for RBCT. Our findings underline the need for further studies to define the optimal management of anemia in SAH patients.

## Introduction

Patients’ age, the severity of initial bleeding, aneurysm rerupture and occurrence of delayed cerebral ischemia (DCI) in aneurysmal subarachnoid hemorrhage (SAH) are major contributors to poor outcome^1^. Moreover, the morbidity and mortality of SAH might be influenced by a multitude of medical conditions and complications during the critical care period including the occurrence of systemic infections and disturbances in blood composition and pressure^2–5^.

Anemia is a common finding in SAH patients and has also been linked to an increased risk of poor outcome^6,7^. Although anemia is an essentially curable condition that can be quickly corrected with blood transfusion, the largest currently available clinical study^8^ showed a better risk–benefit profile with a restrictive transfusion regime, transfusing only at hemoglobin (Hb) values < 7 g/dL. However, this evidence is based on a general critical care population, mostly without neurological symptoms due to acute brain injury. Whether such a low transfusion threshold is also reasonable for SAH patients with compromised cerebrovascular perfusion remains unclear.

In several SAH series, anemia has been reported to correlate with poor outcome and complications such as vasospasm and cerebral infarctions^9–13^. However, a large discrepancy in the reported rates of anemia, mostly retrospective design and small sample sizes of these studies strongly limit the generalizability of the conclusions made by the single articles. Moreover, the authors often used different approaches in the assessment of anemia and goal Hb for red blood cell transfusion (RBCT). In turn, RBCT in SAH has also been associated with poor outcome^14^. As a result, there are still no specific recommendations on anemia management in SAH guidelines^15–17^. This discrepancy is nicely illustrated in a North American study, reporting a range of transfusion thresholds for SAH patients as stated in an expert opinion survey^18^.

We aimed to perform a systematic review and meta-analysis to evaluate the association between anemia with functional outcome and complications of SAH. An emphasis was put on the identification of outcome-relevant anemia severity.

## Methods

This systematic review has been designed and performed according to the PRISMA guidelines^19^. The data that support the findings of this study are available from the corresponding author upon reasonable request.

### Search strategy and selection criteria

We searched five academic databases (PubMed, Scopus, Embase, Cochrane Library and Web of Science) for articles published in one of the eligible languages (English, German, Dutch, Russian, Persian and Turkish) before the twenty third of October 2022, reporting data on anemia in SAH patients (see Supplementary Table S1 for the complete list of search terms). M.S. screened the titles and abstracts of all collected publications. In case of ambiguity about the including a paper, the reviewed article was discussed between M.S. and R.J. and inclusion was cleared. Reference lists of relevant publications were screened for additional articles by M.S. and R.J. in the same manner.

Data from case–control, cross-sectional and longitudinal clinical studies reporting on anemia and/or RBCT in the context of acute SAH were included. The studies were considered eligible if they reported on:

- anemia at admission and/or during the first weeks after SAH quantifying it with Hb, red blood cell (RBC) or hematocrit (Hct) values and/or the need for RBCT;
- risk factors for anemia/RBCT at admission and during treatment;
- the association between anemia/RBCT with the functional outcome and clinical course of SAH, impairment of cerebral oxygenation and metabolism, as well development of different systemic and central nervous system complications.

The publications including cases with non-aneurysmal, non-traumatic SAH and those limited to specific selection criteria (such as age or treatment modality) were not excluded from the final selection list. Only the data that could be clearly referred to SAH cases were extracted for further analyses out of the studies reporting on patients with ruptured and unruptured intracranial aneurysms.

Excluded were the studies with mixed cohorts where the data on SAH patients could not be separately collected, small case series (< 20 patients) and case reports, as well as the articles in non-eligible languages and/or without available full-text.

### Data collection

Data extraction from the manuscripts included in the systematic review was performed by M.S. and the quality of all extracted data controlled by R.J. In the case of multiple publications from the same cohort, we included only the one with the most complete data. Studies with partially overlapping cohorts were not excluded from analyses, however, this was mentioned as a limitation.

Included studies contained information on: (a) study and population characteristics; (b) quantitative data on the rate(/severity) of anemia and RBCT at different time points (as reported by the authors, including institution-specific thresholds for RBCT); (c) various demographic, clinical, radiographic, laboratory and other diagnostic and therapeutic characteristics of both patients and SAH severity.

### Quality assessment

For each included study a quality score, based on the Newcastle–Ottawa Scale^20^ (see Supplementary Table S2) was independently calculated by two raters (M.S. and R.J.). Articles scoring seven stars and more were regarded as good quality studies. No study was excluded from further analysis after the quality assessment.

### Study endpoints and data synthesis

As the objective of the present study was the identification of outcome-relevant severity of post-SAH anemia, the following clinical events were selected as primary study endpoints: occurrence of cerebral infarction (and/or ischemia in any brain imaging) and vasospasm (symptomatic, angiographic or ultrasonographic), in-hospital (/30-days) mortality as well as poor outcome at discharge and at follow-up (as specified by the authors). Moreover, the following clinical events were also correlated with anemia severity as secondary endpoints: (a) patients’ demographic characteristics and previous medical history, as well as initial clinical and radiographic severity of SAH as potential predictors of anemia and/or the need for RBCT; (b) impact of anemia and RBCT on the clinical course of SAH assessed with different clinical variables such as the treatment duration, occurrence of specific complications, etc. (see Supplementary Table S3 for the full list of correlations between post-SAH anemia and other recorded variables).

The presence of anemia was analyzed at the following time points—at admission, perioperatively and during the acute course of SAH. All quantifiable data on anemia were converted into Hb values to enable data synthesis of anemia severity and its association with the primary endpoints. For one study based on Hct measurements^21^, the estimation of Hb was performed upon the standard formula (Małgorzata Koperska, MD; https://www.omnicalculator.com/health/hct-hgb). To estimate Hb values from the studies analyzing the impact of RBCT on the primary review endpoints, the corresponding institutional cutoffs for RBCT or, if reported, the mean/nadir Hb values in the RBCT/non-RBCT subgroups were applied^9,12,14,21–34^. In the remaining studies, the severity of anemia could be assessed upon the reported absolute, nadir and mean/median Hb values. The collected data on Hb values were analyzed as continuous or dichotomous variables, as appropriate. Due to the large heterogeneity of reported Hb cutoffs, the dichotomous Hb values were grouped for further analyses as follows: 11.0–11.5 g/dL, 9.0–10.0 g/dL, 8.0–8.5 g/dL, and 7.0 g/dL.

### Statistical analysis

For the analysis between post-SAH anemia and the study endpoints, a formal meta-analysis was performed using Review Manager (version 5.3.5, Nordic Cochrane Centre, Copenhagen, Denmark). To address the assumed heterogeneity, random-effects models of meta-analysis (Mantel–Haenzsel method [M–H]) were used for all comparisons. Slight to moderate heterogeneity was defined as I^2^ ≤ 60% and substantial heterogeneity as I^2^ > 60%. Funnel plot inspection was applied to evaluate potential publication bias in the analysis of the pooled data (see Supplementary Fig. S1 in Online Supplements). The statistical results were reported as stated in the original publications for the correlations which could not be pooled for further meta-analysis (due to data incongruence or analysis based on a single study). Differences with a P-value of < 0.05 were regarded as statistically significant.

### Assessment of the level of evidence

M.S. and M.G. independently evaluated the evidence level for each analyzed association. The assessment was performed according to the GRADE (Grading of Recommendations Assessment, Development and Evaluation) guidelines^35^ and adapted to the scope of the present review (see Supplementary Table S4).

### Ethics approval and consent to participate

Our study did not require an ethical board approval because it concerns a systematic review and meta-analysis of previously published articles. We did not include any patient/personal data from our own or any other institute.

## Results

### Study and population characteristics

**Figure 1:**
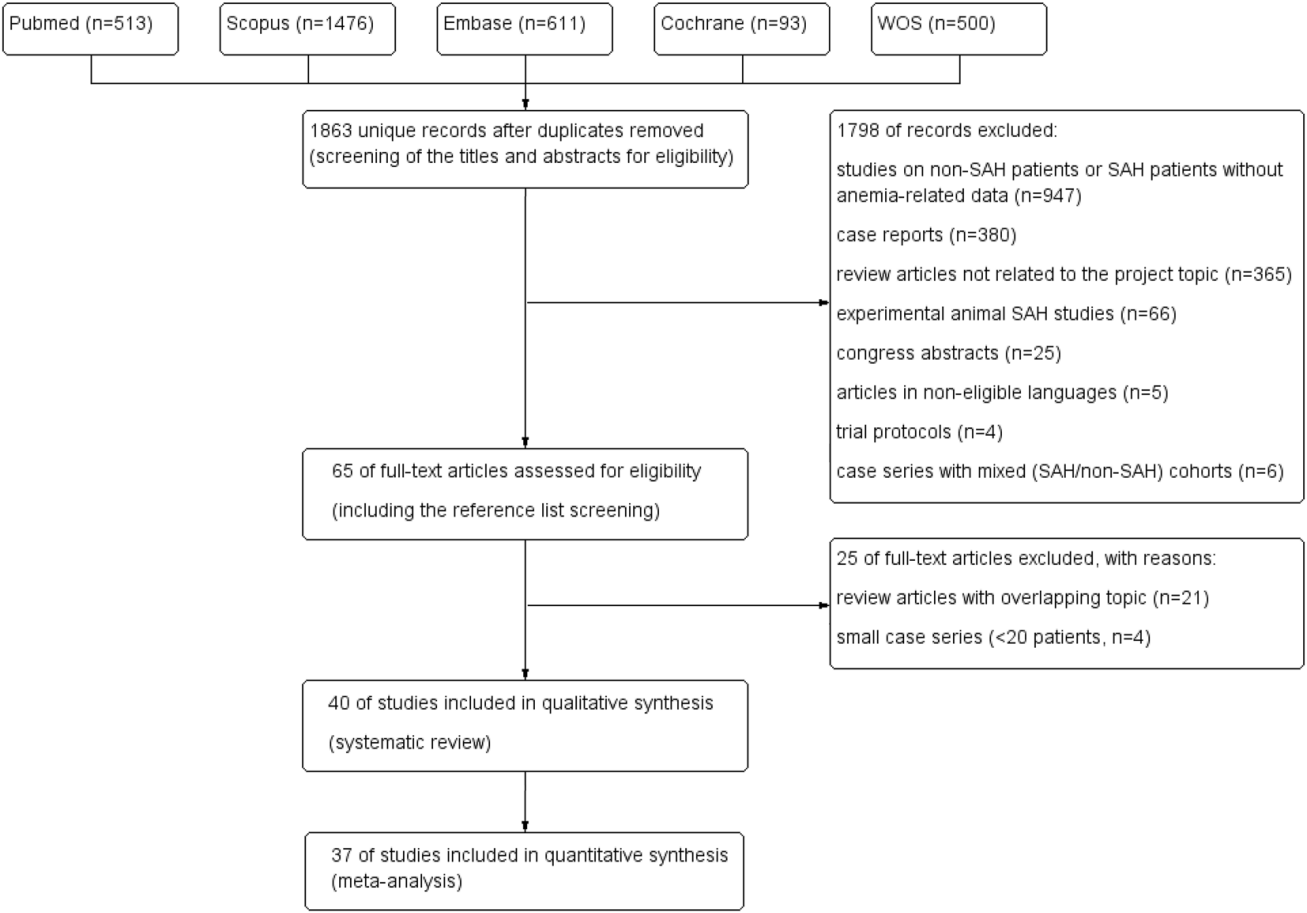
Flow-chart with the selection process of the eligible studies. *SAH* subarachnoid hemorrhage, *WOS* web of Science.

A total number of 1863 original records were identified through the database search. Of these, 40 full-text articles with a total of 14,701 patients treated between 1996 and 2020 were included in the final analysis (mean 445.48 patients per study). The flow chart (Fig. 1) shows the selection process of the eligible studies.

The vast majority of the studies were conducted in the USA (n = 22) and China (n = 9). Along with aneurysmal SAH cases, six studies^10,12,25,26,34,36^ also included patients with non-aneurysmal non-traumatic SAH. Specific selection criteria (such as patients’ age, treatment modality and severity of SAH) were applied in 12 studies^27,32,33,37–45^.

Mean patients’ age in the collected dataset was 55.32 years (95% confidence interval [CI] 55.14–55.49), 63.49% (95% CI 63.43–63.56) were females, 27.37% (95% CI 27.13–27.60) presented with higher initial clinical SAH grade, and 64.73% (95% CI 64.18–65.28) with a higher burden of intracranial hemorrhage. Aneurysm clipping was performed in 52.59% (95% CI 52.17–53.01) of SAH patients. Anemia (as specified by the authors) occurred in 40.76% of SAH individuals during treatment, ranging between 28.3 and 82.6%. There was a large heterogeneity regarding the reported Hb thresholds for RBCT (between 7.0 and 10.0 g/dL, mean: 8.5 g/dL). Accordingly, the rates of RBCT ranged widely between 7.8 and 88.6% (mean 32.07%). Detailed characteristics of the included studies are presented in the Supplementary Table S5.

### Association between anemia severity and the primary review endpoints

**Figure 2:**
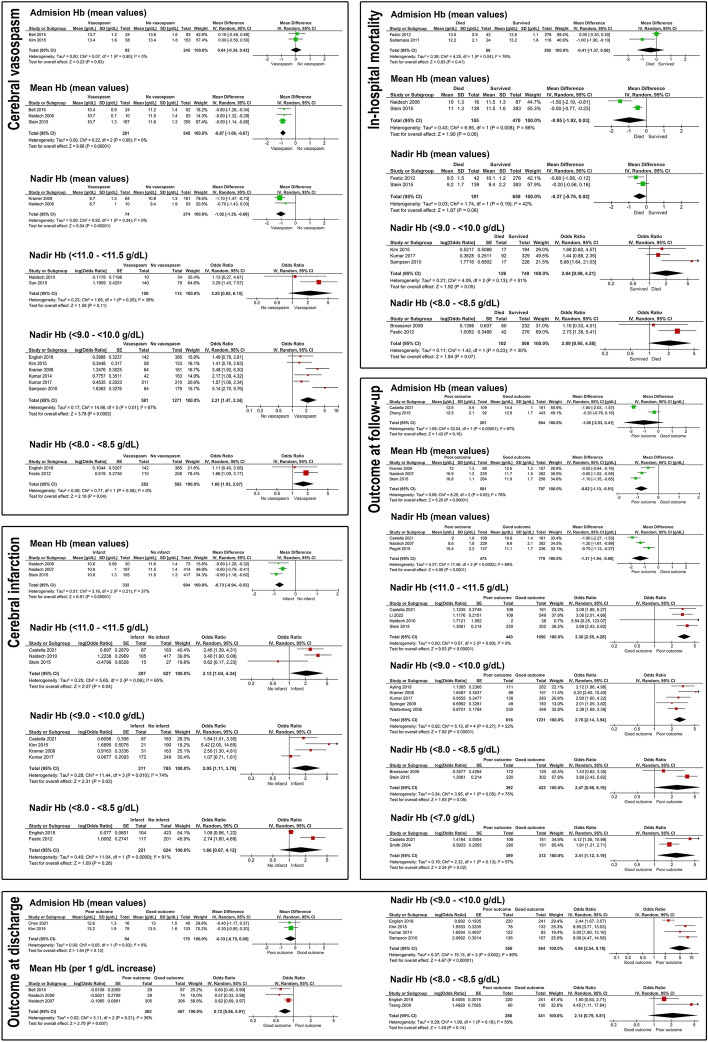
Forest plots analyses for the association between the severity of post-SAH anemia and selected primary endpoints: cerebral vasospasm, cerebral infarction, in-hospital mortality, poor outcome at discharge and at follow-up.

**Figure 3:**
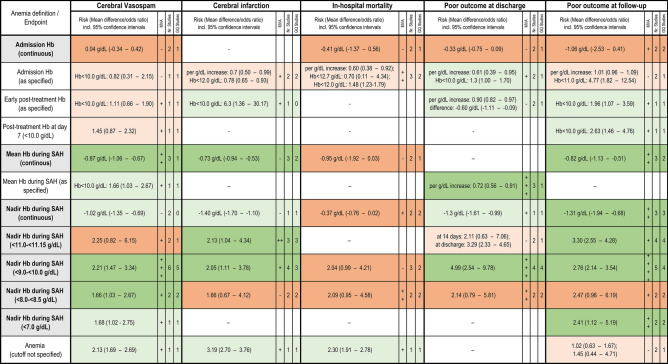
Summary overview of the evidence for the association between anemia severity and primary endpoints. The risk for each primary endpoint is reported for continuous and dichotomous Hb values. Correlations tested in multivariate analysis, the number of studies included per subgroup and the number of good-quality studies are reported. Significant associations reaching the evidence level I-II are marked dark green, the associations with lower evidence—light green. Non-significant findings are marked orange or light orange for associations with an evidence level I–II or lower, respectively. *Hb* Hemoglobin, *SAH* subarachnoid hemorrhage, *GQ* good quality, *MVA* multivariate analysis.

Figure 2 presents the forest plots addressing the impact of anemia severity on the primary endpoints—cerebral vasospasm, cerebral infarction, in-hospital mortality and poor outcome at discharge and follow-up. For a better overview, we also summarized the evidence from the forest plots with those from the remaining studies (Fig. 3). Post-SAH anemia was not associated with the risk of in-hospital mortality. There was no or a merely weak link between early anemia (present at admission and perioperatively) and the primary endpoints. In contrast, anemia developing during SAH treatment (assessed by mean and nadir Hb values) showed significant associations at sufficient evidence level with the risk of cerebral vasospasm, infarction and poor outcome at discharge and follow-up. Significant results were observed already at the early stages of anemia (for nadir Hb 11.0–11.5 g/dL and the risk of cerebral infarcts [odds ratio (OR) 2.13, 95% CI 1.04–4.34, p = 0.04, I^2^ = 65%] and poor long-term outcome [OR 3.30, 95% CI 2.55–4.28, p < 0.0001, I^2^ = 0%]). Moreover, significant associations were also reported for anemia at a nadir Hb 9.0–10.0 g/dL and the risk of cerebral vasospasm (OR 2.21. 95% CI 1.47–3.34, p = 0.0002, I^2^ = 67%), infarction (OR 2.05, 95% CI 1.11–3.78, p = 0.02, I^2^ = 74%, and poor outcome at discharge (OR 4.99, 95% CI 2.54–9.78, p < 0.0001, I^2^ = 80%) and follow-up (OR 2.76, 95% CI 2.14–3.54, p < 0.0001, I^2^ = 22%). A further drop in Hb did not increase the odds ratios for the primary endpoints.

The analysis of nadir Hb during SAH as a continuous variable showed stronger Hb decline in SAH individuals with cerebral vasospasm (∆ = 1.02 g/dL, 95% CI 0.69–1.35, p < 0.0001, I^2^ = 0%), cerebral infarction (∆ = 1.4 g/dL, 95% CI 1.1–1.7, p = 0.001, based on single study^23^), poor outcome at discharge (∆ = 1.3 g/dL, 95% CI 0.99–1.61, p < 0.0001, based on single study^23^) and follow-up (∆ = 1.31 g/dL, 95% CI 0.68–1.94, p < 0.0001, I^2^ = 89%) than in the comparison group without these complications. The decline of mean Hb values during SAH was also associated with the primary endpoints—cerebral vasospasm (∆ = 0.87 g/dL, 95% CI 0.67–1.06, p < 0.0001, I^2^ = 0%), cerebral infarction (∆ = 0.73 g/dL, 95% CI 0.53–0.94, p < 0.0001, I^2^ = 37%), and poor outcome at long-term follow-up (∆ = 0.82 g/dL, 95% CI 0.51–1.13, p < 0.0001, I^2^ = 76%).

### Association between anemia and the need for RBCT with the secondary review endpoints

#### Early anemia and the need for early (perioperative) RBCT

OF the analyzed patients’ characteristics, only female sex was identified as a predictor of early anemia after SAH. Patients with a higher clinical and radiographic grade of SAH were more likely to present with anemia at admission and require RBCT during aneurysm clipping. Perioperative RBCT was more common in individuals with longer duration of aneurysm surgery and intraoperative aneurysm rupture (see Supplementary Table S6). Furthermore, early anemia was associated with the risk of acute seizures.

#### Anemia and the need for RBCT during the acute SAH course

**Figure 4:**
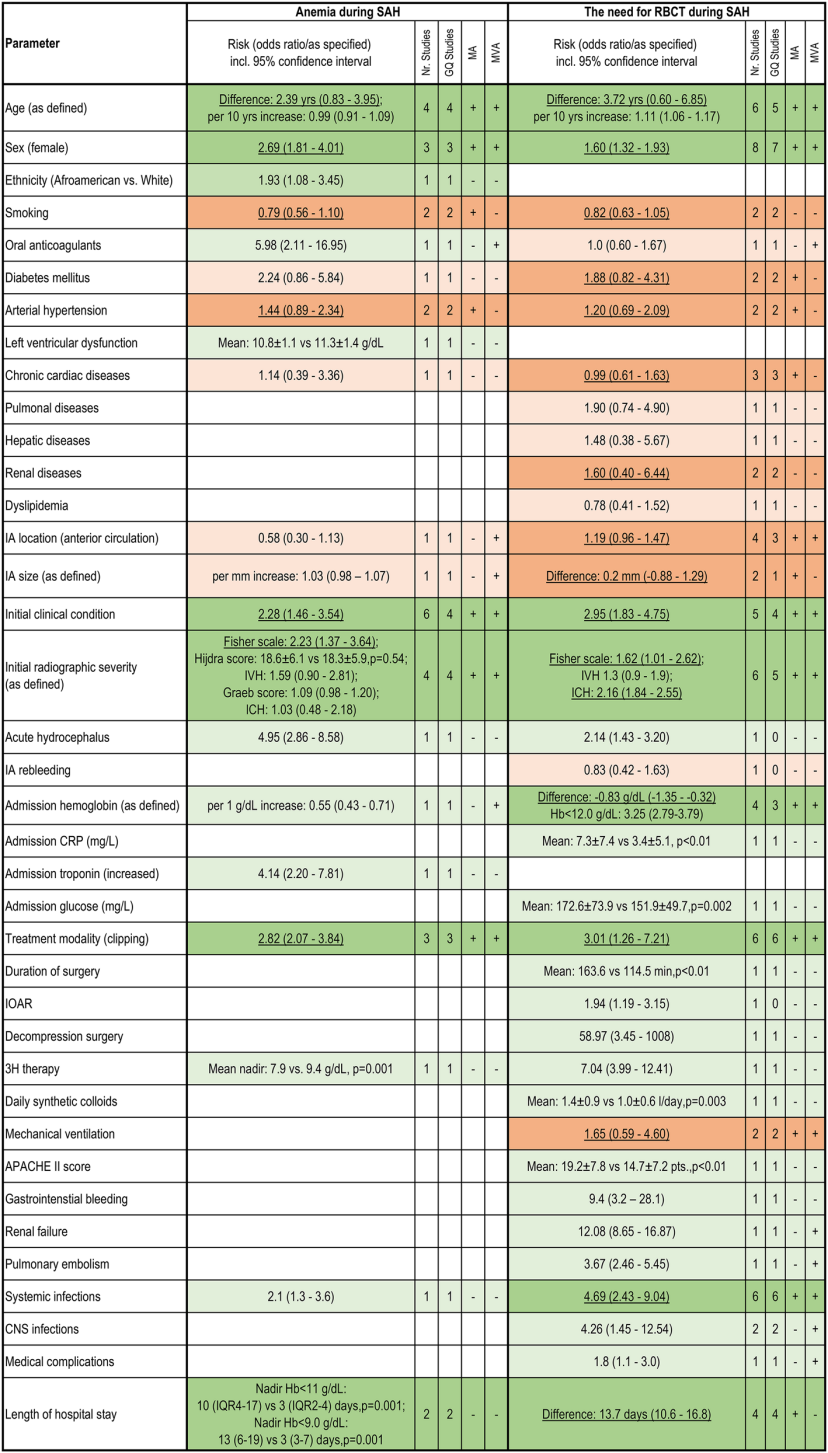
Summary overview of the evidence for the association between anemia and other study endpoints. The individual parameters are set out against the risk of developing anemia during SAH and the need for RBCT during the acute course. Correlations tested in multivariate analysis, the number of good-quality studies and the total number of studies included in the analyses are reported. Significant findings with sufficient (level I–II) und weak (level III–IV) evidence levels are marked in dark and light green, respectively. Fittingly, nonsignificant findings are marked dark (evidence level I–II) and light orange (lower evidence), respectively. *SAH* subarachnoid hemorrhage, *Hb* Hemoglobin, *RBCT* red blood cell transfusion, *GQ* good quality, *MVA* multivariate analysis, *MA* meta-analysis, *CNS* central nervous system, *IOAR* intraoperative aneurysm rupture, *IA* intracranial aneurysm, *3H-therapy* triple H (hypervolemia, hypertension, hemodilution). The results depicted in the forest plot analyses are underlined.

Older individuals and females were at higher risk for anemia and the need for RBCT. Lower evidence was found for the predictive value of ethnicity, use of oral anticoagulants and presence of left ventricular dysfunction (see Fig. 4 with the appropriate analyses and the assessment of the evidence level). SAH patients with poor initial clinical and radiographic condition and certain laboratory findings (lower Hb and higher c-reactive protein, glucose and troponin at admission) were more prone to anemia and/or the need for RBCT during SAH. Finally, aneurysm clipping, duration of surgery, intraoperative aneurysm rupture, need for decompressive surgery and 3H-therapy, APACHE II score, gastrointestinal bleeding, occurrence of systemic and central nervous system infections, and length of hospital stay were associated with post-SAH anemia and the need for RBCT.

## Discussion

This meta-analysis and systematic review provides an overview of the available data in medical literature on anemia after SAH. We found early anemia to be of limited clinical relevance for SAH patients, whereas anemia occurring during the further course of disease was strongly associated with multiple complications and an overall poor outcome in patients with SAH.

### Impact of anemia severity on the clinical course and outcome of SAH

Several retrospective studies published during the last two decades showed significant associations between anemia and/or RBCT and outcome of SAH^14,31^. However, there is still no consensus on optimal Hb values after aneurysm rupture. As a result, institutional policies on RBCT strongly vary throughout neurovascular centers^18^. A large systematic review on anemia and transfusion after SAH by Le-Roux in 2011 concluded that a restrictive RBCT regime based on the results of the Transfusion Requirements Critical Care Trial does not apply to SAH patients and that randomized trials to address the role of RBCT in SAH are required^46^. The protocol of a pilot randomized controlled SAHaRA-trial (Aneurysmal SubArachnoid Hemorrhage—Red Blood Cell Transfusion And Outcome) comparing liberal with restrictive RBCT strategy after SAH was published recently^47^. However, neither preliminary nor final trial results have been reported yet.

The evidence from small observational clinical and experimental studies indicates potential benefits of a higher Hb goal for cerebral oxygenation of the damaged brain after aneurysm rupture. Our systematic review found recent studies that reported on significant associations between Hb values and local oxygen tension measurements, tissue hypoxia, metabolic distress and cell-energy dysfunction in SAH patients^42–44^. In vulnerable brain regions, an increase of the global cerebral blood flow, oxygen extraction fraction and cerebral metabolic rate for oxygen were shown after RBCT^44^. These findings speak in favor of a liberal RBCT regime. On the other side, higher Hb values may reduce cerebral blood flow (due to autoregulatory vasoconstriction) and increase blood viscosity^46^. In addition, a higher risk of organ dysfunction and mortality in individuals receiving RBCT was shown for general critical care^48,49^ and SAH patients^14^. This association might be, at least partially, conditioned by an inflammatory and immunomodulatory response of recipients to allogenic RBCT^50,51^. Therefore, the question of the optimal Hb values for SAH is not solved yet.

In this systematic review and meta-analysis based on 37 studies published between 2004 and 2022, almost half of the SAH patients developed anemia during the acute phase treatment. On average, every third SAH patient required RBCT. Of note, the rates of RBCT in the cohorts varied strongly (7.8–88.6%) throughout the studies. Although anemia rates were also different in the single cohorts, this vast discrepancy in RBCT frequency was, to a considerable extent, related to different transfusion thresholds. In line with the previously mentioned North American survey on RBCT practices^18^, nadir Hb values necessary for RBCT varied between 7.0 and 10.0 g/dL. In about half of the studies reporting on RBCT threshold, a restrictive regime of RBCT in SAH patients was utilized, whereas a more liberal approach to RBCT policy (particularly, for individuals at risk of DCI) was reported in five studies from the USA.

Although this discrepancy and heterogeneity in the collected data complicated further data synthesis and analysis, we could derive several relevant findings from this meta-analysis which might help in further elaboration of specific guidelines for RBCT in SAH patients. First, anemia at the onset of SAH showed little to no impact on the further course of SAH. In contrast, even slight anemia (11.0–11.5 g/dL) occurring during SAH showed significant correlations with the risk of cerebral infarction and poor outcome at discharge and follow-up. The most substantial evidence concerning the primary study endpoints was observed for moderate anemia at nadir Hb values between 9.0 and 10.0 g/dL. There was no increase in the risk burden for poor outcome with further increasing severity of post-SAH anemia. Finally, the observed effect of anemia on SAH morality was marginal.

In virtue of the clinical relevance of post-SAH anemia, knowledge of risk factors for the occurrence of anemia is essential for risk-adapted management of SAH patients. A variety of predisposing factors for the development of anemia after aneurysm rupture were documented in the reviewed studies. The most relevant risk factors for post-SAH anemia are older age, female sex, higher initial severity of SAH, the burden of surgical interventions and occurrence and treatment of cerebral vasospasm. At the same time, the above-mentioned risk factors for post-SAH anemia are also acknowledged as strong contributors to the risk of DCI and poor outcome after SAH^52,53^. Accordingly, the avoidance of severe anemia in these high-risk SAH individuals, particularly during the first 2 weeks after SAH, by utilizing a more liberal RBCT strategy than currently practiced in the majority of intensive care units (RBCT at Hb decrease < 7.0 g/dL) might be worthwhile. As our systematic review showed the strongest association between anemia and poor SAH outcome at Hb values between 9.0 and 10.0 g/dL, this Hb range might present an appropriate Hb threshold justifying RBCT in SAH patients at high risk of cerebral ischemia and poor outcome.

Of note, our systematic review also confirmed the previous observations from single studies showing a higher probability of complications and longer hospital stay in SAH individuals receiving RBCT. Whether this association is related to RBCT itself, or to the risk factors necessitating RBCT should be clarified in further studies, as only a handful of the reported analyses adjusted their results to the relevant confounders.

### Limitations

The limitations related to the large heterogeneity of the collected data have already been mentioned. Substantial differences in the definitions of anemia and RBCT policies across the studies did not allow comparisons to be fully extendable to the pooled data, necessitating the generalization, conditional conversion and grouping of data as it has been stated in the methods section and also described in the Supplementary Table S7. Furthermore, there was some geographic overrepresentation of the cohorts from the USA and China, together making up about three-quarters of all studies included in the analysis. Despite these limitations, this systematic review offers a comprehensive and objective summary of the evidence on anemia and SAH that could be useful to set up general guidelines for the treatment of anemia in the context of SAH.

## Conclusions

This systematic review and meta-analysis summarizes the available literature on the occurrence and treatment of anemia in SAH patients. Presence of anemia at the onset of SAH seems to be of limited relevance in its further course. In contrast, even slight anemia (nadir Hb < 11.0–11.5 g/dL) occurring during SAH is significantly associated with the risk of cerebral infarction and poor outcome at discharge and follow-up. The most substantial evidence for SAH outcome was observed for nadir Hb values between 9.0 and 10.0 g/dL. Further studies are required to define the optimal management of anemia in SAH.

## Supplementary Information

Supplementary Information 1.

Supplementary Information 2.

## Data Availability

Available upon reasonable request via email to the first author (Maryam.said@uk-essen.de).
