# Supplementary material for: Systematic review and meta-analysis of outcome-relevant anemia in patients with subarachnoid hemorrhage
Source: Sci Rep. 2022 Dec 1;12:20738. doi: 10.1038/s41598-022-24591-x (PMC9715711; doi:10.1038/s41598-022-24591-x)
Supplement: Supplementary file 2 — Supplementary Information 2. [file 41598_2022_24591_MOESM2_ESM.docx]

**SUPPLEMENTARY MATERIALS**

**Supplementary Figure S1:** Funnel plots of the performed meta-analyses regarding the association between anemia and SAH outcome endpoints graphically showing the presence of publication bias.

| 1. **The value of anemia for the long-term functional outcome after SAH** | |
| --- | --- |
| *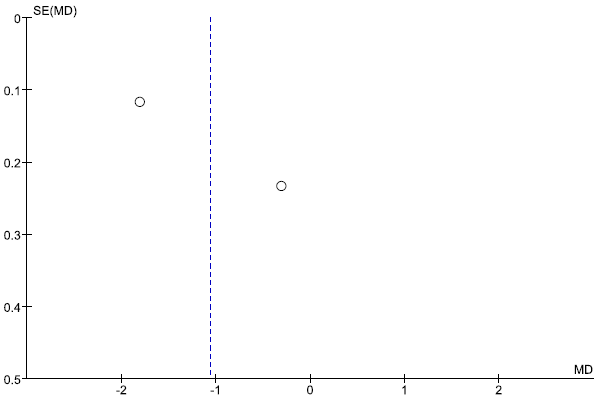*  *Admission Hb (continuous assessment)* | *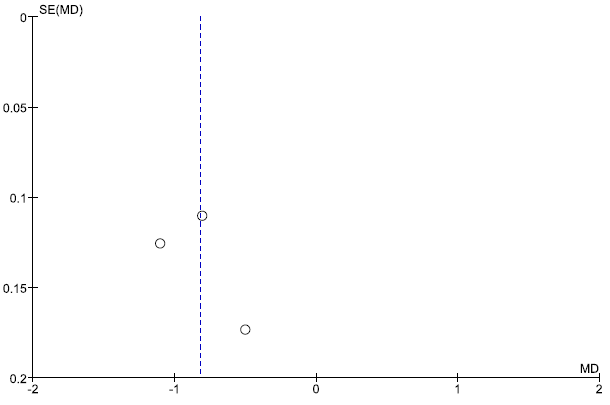*  *Mean Hb during SAH (continuous assessment)* |
| *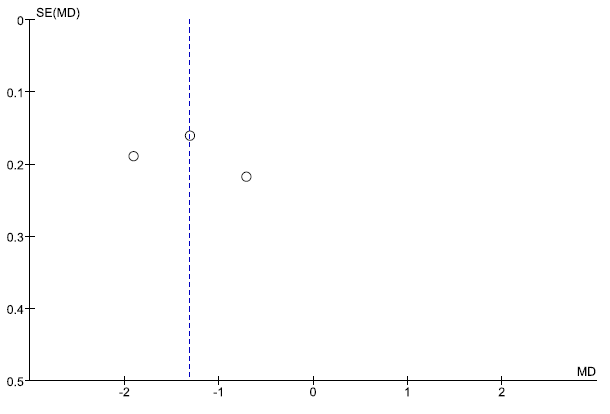*  *Nadir Hb during SAH (continuous assessment)* | *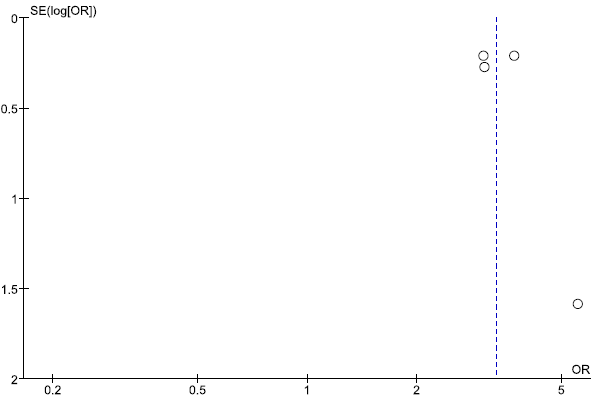*  *Nadir Hb during SAH or RBCT at <11.0 – 11.5 g/dL* |
| *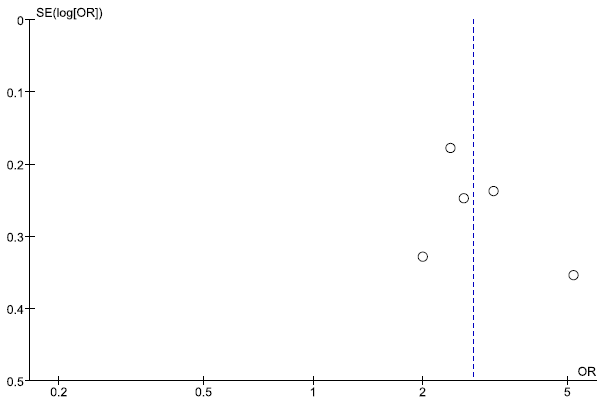*  *Nadir Hb during SAH or RBCT at <9.0 – 10.0 g/dL* | *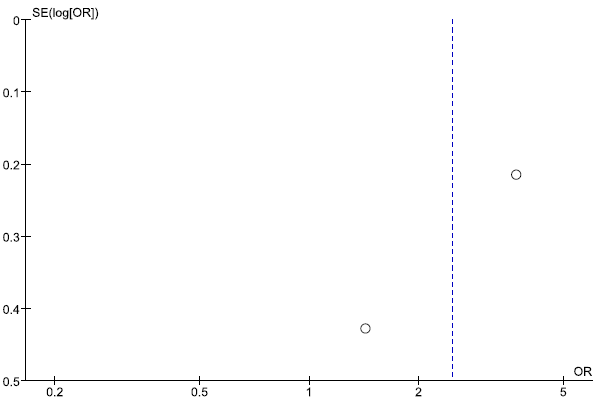*  *Nadir Hb or RBCT at <8.0 – 8.5 g/dL* |
| *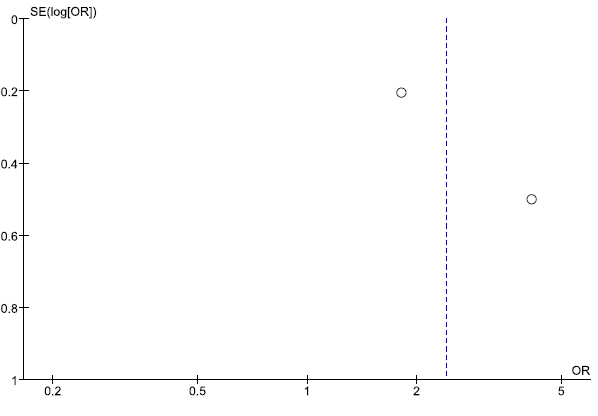*  *Nadir Hb or RBCT at <7.0 g/dL* | |
| 1. **The value of anemia for the functional outcome after SAH at discharge (short-term outcome)** | |
| *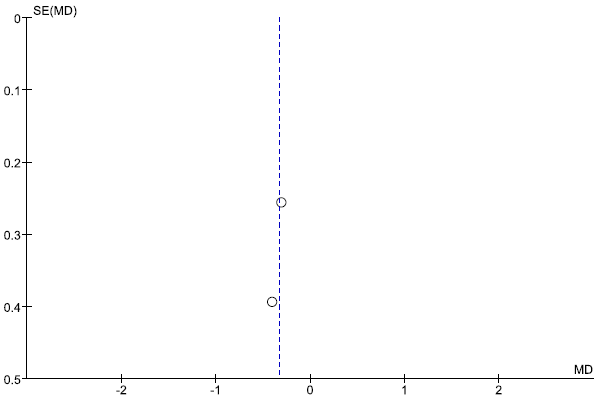*  *Admission Hb (continuous assessment)* | *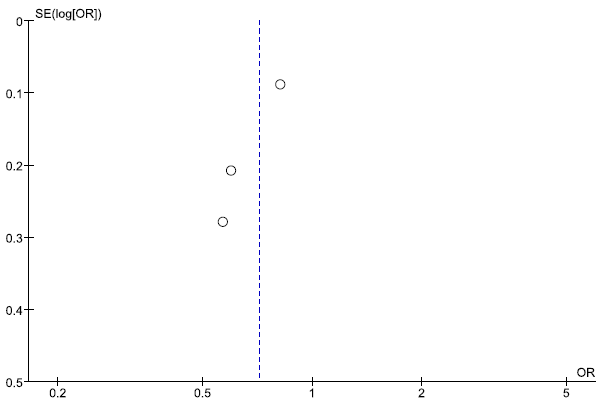*  *Mean Hb during SAH (continuous assessment)* |
| *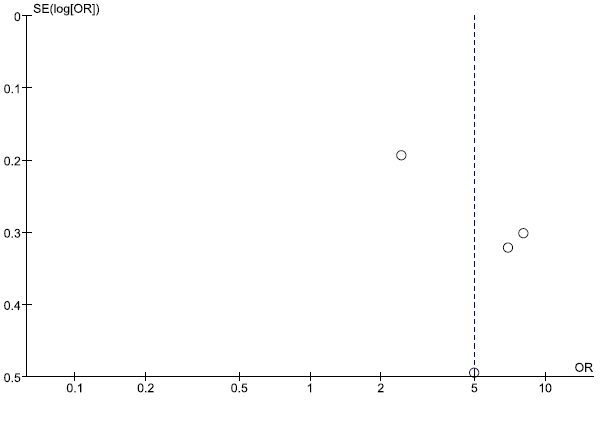*  *Nadir Hb during SAH or RBCT at <9.0 – 10.0 g/dL* | *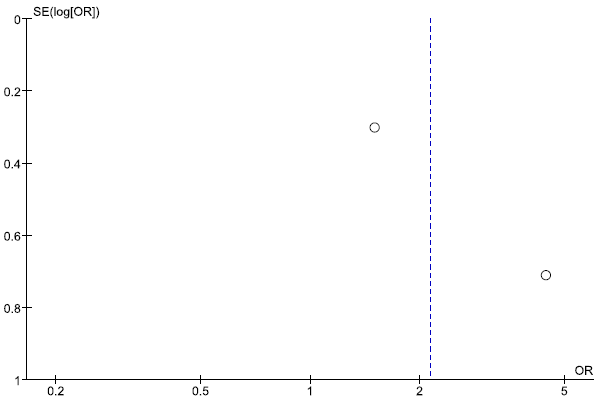*  *Nadir Hb or RBCT at <8.0 – 8.5 g/dL* |
| 1. **The value of anemia for in-hospital (or 30-days) mortality after SAH at discharge** | |
| *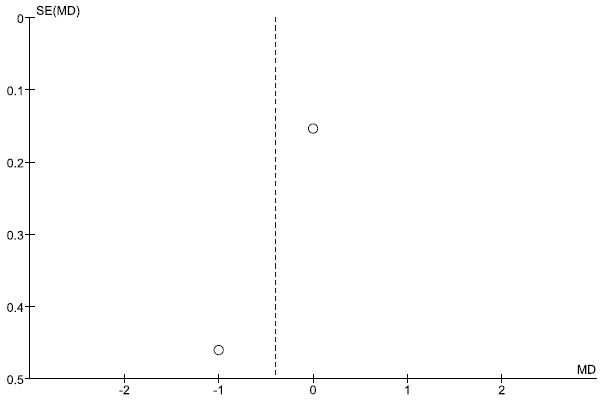*  *Admission Hb (continuous assessment)* | *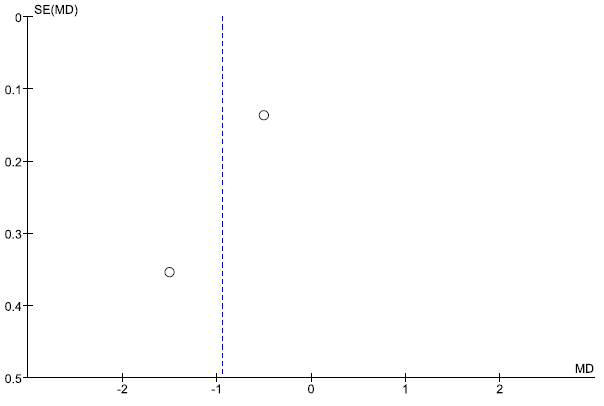*  *Mean Hb during SAH (continuous assessment)* |
| *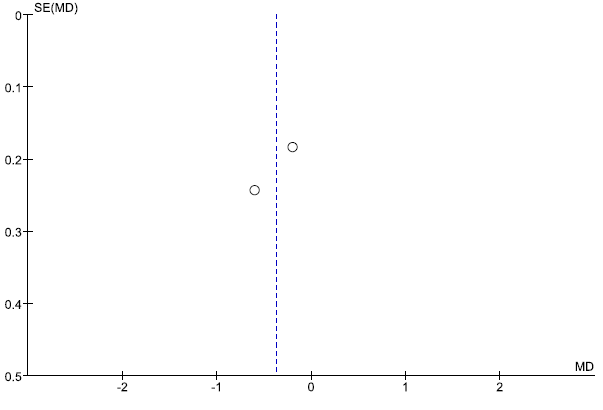*  *Nadir Hb during SAH (continuous assessment)* | *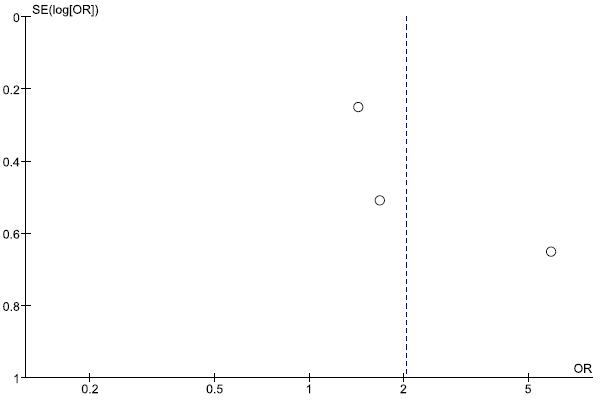*  *Nadir Hb during SAH or RBCT at <9.0 – 10.0 g/dL* |
| *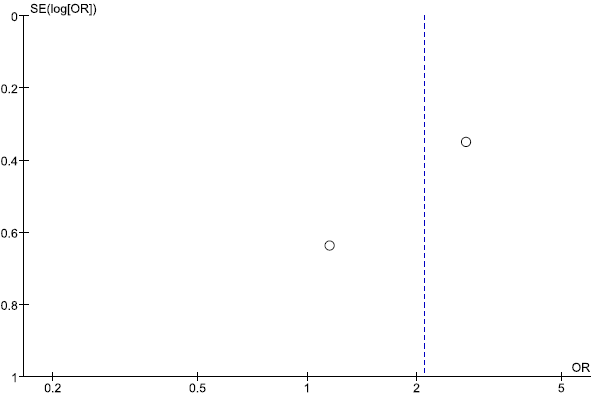*  *Nadir Hb or RBCT at <8.0 – 8.5 g/dL* | |
| 1. **The value of anemia for the risk of cerebral infarction after SAH** | |
| *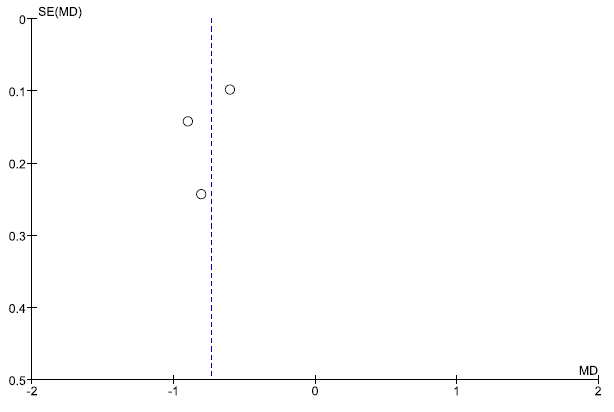*  *Mean Hb during SAH (continuous assessment)* | *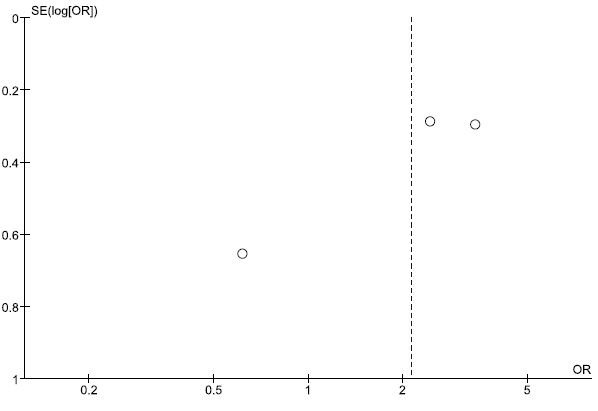*  *Nadir Hb during SAH or RBCT at <11.0 – 11.5 g/dL* |
| *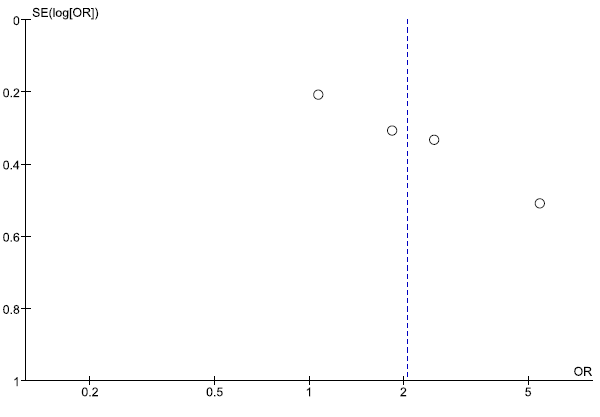*  *Nadir Hb during SAH or RBCT at <9.0 – 10.0 g/dL* | *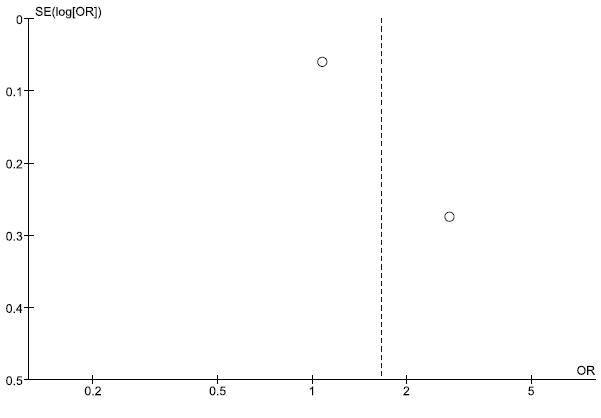*  *Nadir Hb or RBCT at <8.0 – 8.5 g/dL* |
| 1. **The value of anemia for the risk of cerebral vasospasm after SAH** | |
| *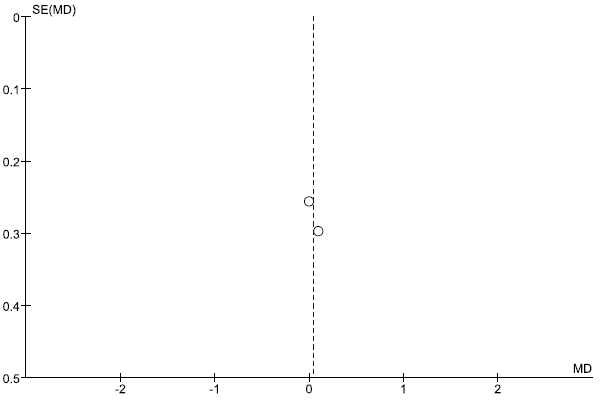*  *Admission Hb (continuous assessment)* | *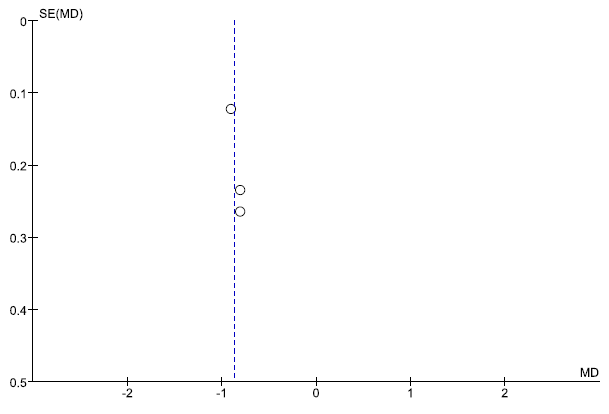*  *Mean Hb during SAH (continuous assessment)* |
| *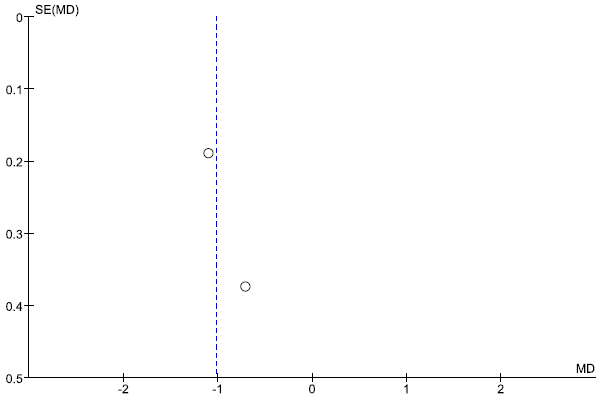 Nadir Hb during SAH (continuous assessment)* | *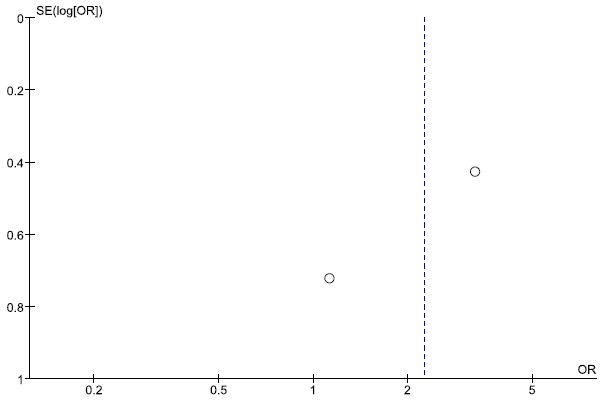*  *Nadir Hb during SAH or RBCT at <11.0 – 11.5 g/dL* |
| *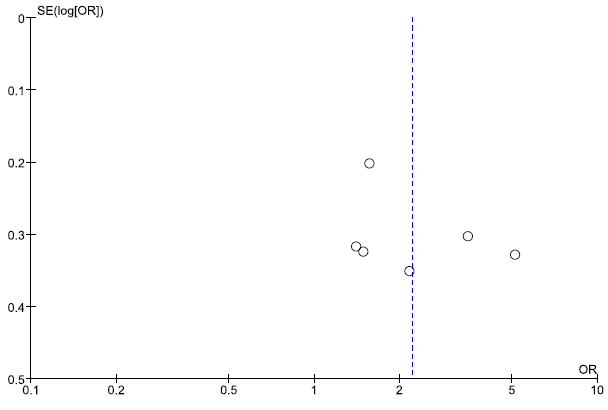*  *Nadir Hb during SAH or RBCT at <9.0 – 10.0 g/dL* | *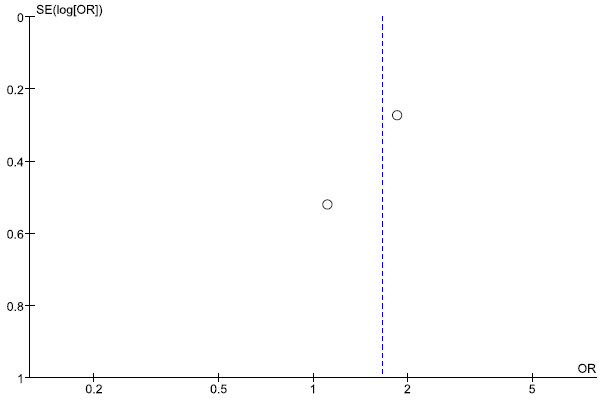*  *Nadir Hb or RBCT at <8.0 – 8.5 g/dL* |

**Supplementary Table S1**: A Full list of search terms and screened academic databases

| **Academic databases** | **Query text** | **Search results:** |
| --- | --- | --- |
| Scopus | (TITLE-ABS-KEY ("subarachnoid hemorrhage") AND TITLE-ABS-KEY (anemia OR "red blood cell" OR hemoglobin OR erythrocyte OR "blood transfusion") AND TITLE-ABS-KEY (patient)) | 1476 |
| Pubmed | "subarachnoid hemorrhage" and (anemia or "red blood cell" or hemoglobin or erythrocytes or "blood transfusion") and patient | 513 |
| Embase |  | 611 |
| Web of Science |  | 500 |
| Cochrane |  | 93 |
| Total number of unique records from all five databases: | | 1863 |
| Query date: | | October 23, 2022 |

**Supplementary Table S2**: The Newcastle-Ottawa Quality Assessment Score of the included studies. A study can be awarded a maximum of one star for each numbered item within the Selection and Outcome categories. A maximum of two stars can be given for Comparability. Studies scoring seven stars and more are considered good quality studies.

| **Selection:** |
| --- |
| 1) Representativeness of the exposed cohort  a) Consecutive eligible SAH cases were selected. **🟑**  b) Not satisfying requirements, or not stated.  2) Selection of the non-exposed cohort  a) drawn from the same SAH population as the exposed cohort **🟑**  b) drawn from a different SAH population  c) no description of the derivation of the non-exposed cohort  3) Ascertainment of exposure  a) secure record **🟑**  b) no description  4) Demonstration that outcome of interest was not present at start of study  a) yes **🟑**  b) no |
| **Comparability** |
| 1) Comparability of cohorts on the basis of the design or analysis  a) study controls for baseline SAH severity and functional outcome **🟑**  b) study controls for any additional confounders (adverse events /complications during SAH) **🟑** |
| **Outcome** |
| 1) Assessment of outcome  a) independent blind assessment (outcome values reported / clear definitions) **🟑**  b) record linkage **🟑**  c) no description  2) Was follow-up long enough for outcomes to occur  a) yes (repetitive anemia assessment during hospital stay) **🟑**  b) no or not stated.  3) Adequacy of follow up of cohorts  a) complete follow up data - all subjects accounted for **🟑**  b) subjects lost to follow up unlikely to introduce bias – lost to follow-up <15%, or description provided of those lost) **🟑**  c) higher rate of missing follow-up and no description of those lost  d) no statement. |

**Supplementary Table S3:** Full list of correlations between post-SAH anemia and different demographic, clinical, radiographic and laboratory patients’ and SAH characteristics (for each analysis group, number of available studies is given in parenthesis)

1. **Long-term functional outcome (n=18)**
   1. Continuous assessment
      1. Admission Hb (Castella2021; Loustarinen2015; Zhang2019)
      2. Mean Hb during SAH: (Kramer2009; Naidech2007; Stein2015)
      3. Nadir Hb during SAH: (Castella2021; Naideich2007; Pegoli2015)
   2. Dichotomized assessment:
      1. Admission Hb <11 (Castella2021)
      2. Post-treatment Hb@d2 <10.0 (Ayling2018)
      3. Post-treatment Hb@d7 <10.0 (Ayling2018)
      4. Mean Hb <11.1 (Stein2015)
      5. Nadir Hb <12.0 (Li2022a, Li2022b)
      6. Nadir Hb <11.0 (Castella2021)
      7. Nadir Hb <10 (Ayling2018; Kramer2009)
      8. Nadir Hb<9.0 (Springer2009; Wartenberg2006)
      9. Not defined cutoff (Qi2021)
      10. RBCT
          1. Cutoff <11.5 (Naidech2010)
          2. Cutoff <10.0 (Kumar2017)
          3. Cutoff <9.5 (Kramer2008)
          4. Cutoff <8.3 (Broessner2009; Stein2015)
          5. Cutoff <7.0 (Castella2021; Smith2004)
          6. Not defined (Loustarinen2015 – intra-OP-RBCT!!!; Pegoli2015)
2. **Poor Outcome at Discharge (n=10)**
   1. Continuous assessment
      1. Admission Hb (Chen2021; Kim2015; Kumar2014; Naidech2006)
      2. First post-treatment Hb (Chen2021; Kim2015)
      3. Mean Hb during SAH: (Bell2015; Naidech2006; Naidech2007)
      4. Nadir Hb during SAH: (Naidech2007)
   2. Dichotomous assessment:
      1. Nadir Hb <12.0 (Li2022b)
      2. Nadir Hb <10.0 (English2018)
      3. RBCT
         1. Cutoff <11.5 (Naidech2010)
         2. Cutoff <10.0 (Kumar2014; Naidech2006; Sampson2010)
         3. Cutoff <8.0 (English2018; Tseng2008; Naidech2007)
         4. Cutoff <9.0-10.0 (Kim2015)
3. **Mortality at Discharge / 30-Days mortality (n=9)**
   1. Continuous assessment:
      1. Admission Hb (Festic2012; Naidech2006; Sadamasa2011)
      2. Mean Hb during SAH (Naidech2006; Stein2015)
      3. Nadir Hb during SAH: (Festic2012; Stein2015)
   2. Dichotomous assessment:
      1. Admission Hb <12.7 (Sadamasa2011)
      2. Admission Hb <12.0 (Schmitt2022)
      3. Nadir Hb <10.0 (Sampson2010)
      4. RBCT:
         1. Cutoff <10.0 (Kumar2017)
         2. Cutoff between <9.0 and <10.0 (Kim2015)
         3. Cutoff <8.5 (Festic2012)
         4. Cutoff <8.3 (Broessner2009)
         5. Not defined (Schmitt2022)
4. **Infarct Risk (n=13)**
   1. Continuous assessment
      1. Admission Hb (Naidech2006; Naidech2007)
      2. Post-treatment Hb (DaSilva2017)
      3. Mean Hb during SAH (Naidech2006; Naidech2007; Stein2015)
      4. Nadir Hb during SAH (Naidech2007)
   2. Dichotomous assessment
      1. Admission Hb <12.0 (Schmitt2022)
      2. Nadir Hb <11.0 (Castella2021)
      3. Nadir Hb <10.9 (Stein2015)
      4. Nadir Hb <10.0 (Kramer2008)
      5. First postoperative Nadir Hb <10.0 (DaSilva2017)
      6. Nadir Hb <9.0 (Castella2021)
      7. RBCT
         1. Cutoff <11.5 (Naidech2010)
         2. Cutoff <10.0 (Kumar2017)
         3. Cutoff between <9.0-<10.0 (Kim2015)
         4. Cutoff <9.5 (Kramer2008)
         5. Cutoff <9.0 (Oddo2009; Castella2021)
         6. Cutoff <8.5 (Festic2012)
         7. Cutoff between <8.0-<10.0 (Naidech2007)
         8. Cutoff <8.0 (English2018)
         9. Not defined (Schmitt2022)
5. **Vasospasm (angiographic/symptomatic/clinical/TCD-based) (n=16)**
   1. Continuous assessment
      1. Admission Hb (Bell2015; Kim2015)
      2. Mean Hb during SAH (Bell2015; Naidech2006; Stein2015)
      3. Nadir Hb during SAH (Kramer2009; Naidech2006)
   2. Dichotomous assessment
      1. Admission Hb <10.0 (Ayling2018)
      2. Post-treatment Hb@d2 <10.0 (Ayling2018; Kim2015)
      3. Post-treatment Hb@d7 <10.0 (Ayling2018)
      4. Mean Hb <10.4 (Stein2015)
      5. Nadir Hb <11.0 (Sun2015)
      6. Nadir Hb <10.0 (English2018; Kramer2008; Sampson2010)
      7. RBCT
         1. Cutoff <11.5 (Naidech2010)
         2. Cutoff <10.0 (Kumar2014; Kumar2017)
         3. Cutoff between <9.0-10.0 (Kim2015)
         4. Cutoff <9.5 (Kramer2008)
         5. Cutoff <8.5 (Festic2012)
         6. Cutoff <8.0 (English2018)
         7. Cutoff <7.0 (Smith2004)
         8. Not defined (Schmitt2022)
6. **Hospital stay duration (n=6)**
   1. Continuous assessment
      1. Mean Hb during SAH (Naidech2007)
      2. Nadir Hb during SAH (Naidech2007)
   2. Dichotomous assessment
      1. Admission Hb <12.0 (Schmitt2022)
      2. Nadir Hb<11.0 (Castella2021)
      3. Nadir Hb<10.0 (Sampson2010)
      4. Nadir Hb <9.0 (Castella2021)
      5. RBCT
         1. Cutoff between <9.0-<10.0 (Kim2015; Levine)
         2. Cutoff <8.3 (Broessner2009)
         3. Not defined (Schmitt2022)
7. **Any systemic infection (n=6)**
   1. Continuous assessment
      1. Admission Hb (Wang2022)
   2. Dichotomous assessment
      1. Admission Hb <12.0 (Schmitt2022)
      2. RBCT
         1. Cutoff between <9.0-<10.0 (Kim2015; Levine2010)
         2. Cutoff <9.5 (Kramer2008)
         3. Cutoff <8.0 (Stein2015)
         4. Not defined (Schmitt2022; Wang2022)
8. **(Predictors of) the need for RBCT (n=13)**
   1. Age (Broessner2009; English2018; Festic2012; Kim2015; Kramer2008; Kumar2014; Kumar2017; Naidech2006; Levine2010; Schmitt2022)
   2. Sex (Broessner2009; English2018; Festic2012; Kim2015; Kumar2014; Kumar2017; Naidech2006; Levine2010; Schmitt2022)
   3. Initial clinical condition (Hunt&Hess/WFNS): (Broessner2009; Festic2012; Kim2015; Kramer2008; Kramer2009; Kumar2014; Kumar2017; Naidech2006; Levine2010)
   4. Initial radiographic severity
      1. Fisher scale, Hijdra Sum score: (English2018; Festic2012; Kim2015; Kramer2008; Kramer2009; Kumar2017; Naidech2006)
      2. ICH (Levine2010)
      3. IVH (Levine2010)
   5. Hydrocephalus (Kumar2017; Levine2010)
   6. Treatment modality (Broessner2009; English2018; Kim2015; Kumar2014; Schmitt2022)
   7. Duration of surgery (Kim2015)
   8. Decompression surgery (Kim2015)
   9. Mechanical ventilation postoperatively (Kim2015; Levine2010)
   10. APACHE II Score (Kim2015)
   11. Oral anticoagulants use (English2018)
   12. Admission Hb (English2018; Festic2012; Kim2015; Kumar2017; Levine2010; Schmitt2022)
   13. Admission CRP (Kim2015)
   14. Admission glucose (Levine2010)
   15. Diabetes (Kim2015; Kumar2014)
   16. Hypertension (Kim2015; Kumar2014)
   17. Cardiac diseases (Kim2015; Kumar2014; Levine2010; Kumar2017)
   18. Smoking (Kumar2014; Kumar2017; Levine2010)
   19. Pulmonal diseases (Kumar2017; Levine2010)
   20. Hepatic diseases (Kim2015)
   21. Renal diseases (Kim2015; Levine2010)
   22. Dyslipidemia (Kim2015)
   23. IA Location (English2018; Kim2015; Kumar2014; Kumar2017; Levine2010)
   24. IA Size (Kim2015; Kumar2017; Levine2010)
   25. IOAR (Kumar2017)
   26. IA rebleeding (Kumar2017; Levine2010)
   27. Gastrointestinal bleeding (Naidech2007)
   28. 3H-therapy (Naidech2007)
   29. Daily synthetic colloids (Tseng2008)
   30. Medical complications (Levine2010)
   31. CNS infection (meningitis/ventriculitis) (Stein2015; Levine2010)
9. **(Predictors of) anemia during SAH (n=9)**
   1. Age (Ayling2018; English2018; Kramer2008; Sampson2010)
   2. Sex (Ayling2018; English2018; Sampson2010; Li2022a)
   3. Ethnicity (Sampson2010)
   4. Smoking (Ayling2018; Sampson2010)
   5. Oral anticoagulants use (English2018)
   6. Diabetes (Sampson2010)
   7. Hypertension (Ayling2018; Sampson2010)
   8. Left ventricular dysfunction (Naidech2007)
   9. Coronary artery disease (Sampson2010)
   10. Initial clinical condition (Ayling2018; Kramer2008; Kramer2009; Naidech2007; Qi2021; Sampson2010)
   11. Initial radiographic severity
       1. Fisher scale (English2018; Kramer2008; Kramer2009; Sampson2010)
       2. Presence of IVH (Ayling2018)
       3. Graeb Score IVH severity (Ayling2018)
       4. Presence of ICH (Ayling2018)
       5. Hijdra score (Ayling2018)
   12. Treatment modality (Ayling2018; English2018; Sampson2010; Li2022b)
   13. Hydrocephalus (Sampson2010)
   14. Admission Hb (English2018)
   15. IA location (English2018)
   16. IA size (English2018)
   17. 3H-Therapy (Naidech2007)
   18. Troponin elevation (Sampson2010)
   19. SIRS score (Sampson2010)
10. **(Predictors of) early anemia after SAH (n=8)**
    1. Age (Ayling2018; Sadamasa2011)
    2. Sex (Ayling2018)
    3. Smoking (Ayling2018)
    4. Hypertension (Ayling2018)
    5. Initial clinical condition (Ayling2018; Broessner2009; Naidech2007; Seicean2015)
    6. Initial radiographic severity
       1. Fisher grade (Sadamasa2011)
       2. Presence of IVH (Ayling2018)
       3. Graeb Score severity of IVH (Ayling2018)
       4. Hijdra sum score (Ayling2018)
    7. Treatment modality (Ayling2018)
    8. IA size (Ayling2018)
    9. Hydrocephalus (Zhang2019)
    10. Acute seizures (Wang2019; Zheng2019)
11. **(Predictors of) peri-/intraoperative RBCT (n=4)**
    1. Age (Chen2021)
    2. Sex (Chen2021; Loustarinen2015)
    3. BMI (Chen2021)
    4. Hypertension (Chen2021)
    5. Initial clinical condition (Chen2021; Loustarinen2015; Seicean2015; Yee2017)
    6. Initial radiographic severity:
       1. Fisher scale: (Chen2021; Loustarinen2015)
       2. Presence of IVH (Yee2017)
       3. Presence of ICH (Yee2017)
    7. IA location (Loustarinen2015)
    8. IA size (Chen2021; Loustarinen2015)
    9. Admission Hb (Chen2021)

11.10 IOAR (Chen2021; Loustarinen2015)

1. Duration of surgery (Chen2021)

**Supplementary Table S4:** Estimation of the Level of Evidence for the associations between post-SAH anemia and other parameters

| **Class** | **Definition** |
| --- | --- |
| I | **High quality**  Data from at least:   1. three good quality studies included in a meta-analysis AND 2. All studies adjusted their results for relevant confounders. 3. Non-conflicting results from good quality studies |
| II | **Moderate quality**  Data from at least:   1. two separate cohorts allowing data pooling and meta-analysis AND 2. one of the studies of good quality OR a study adjusting their results for relevant confounders. |
| III | **Low quality**  Data from at least two separate cohorts allowing data pooling and meta-analysis |
| IV | **Very low quality**  Any lower evidence. |

**Supplementary Table S5:** Baseline characteristics of the studies included in the systematic review

| **Publication (First author & year)** | **Country / Region** | **Number of patients** | **NOS** | **Specific selection** | **Mean / Median Age (years)** | **Females (%)** | **High H&H / WFNS (%)** | **High Fisher (%)** | **Clipping (%)** | **DCI / VS (%)** | **Anemia Rate (%)** | **RBCT threshold (g/dL)** | **RBCT rate (%)** | **Anemia at admission** | **Anemia during SAH** | **Repetitive Sampling** | **Assessed variable** | **Assessment type** |
| --- | --- | --- | --- | --- | --- | --- | --- | --- | --- | --- | --- | --- | --- | --- | --- | --- | --- | --- |
| Ayling 2018 | Israel, North America & Europe | 413 | 9 | - | 51 | 71 | 27 | n/r | 45 | n/r | 32 | 10 | n/r | Yes | Yes | Yes | Hb | Dich. |
| Bell 2015 | USA | 116 | 7 | - | 53.8 | 71 | 2 (med) | 3 (med) | n/r | 21 | n/r | 8.4 | 12.1 | Yes | Yes | Yes | Hb | Cont. |
| Broessner 2009 | Austria | 292 | 9 | aSAH + naSAH | 54.5 | 60 | 25 | n/r | 15.8 | n/r | n/r | 8.3 | 27.1 | Yes | Yes | Yes | Hb | Cont. |
| Castella 2021 | Belgium | 270 | 9 | aSAH + naSAH | 55 | 55 | 34 | 89 | 31 | n/r | 61 | 7 | 7.8 | Yes | Yes | Yes | Hb | Dich. & cont. |
| Chen 2021 | China | 62 | 6 | clipping | 55 | 58.1 | 56.5 | 54.8 | 100 | n/r | n/r | 7 | 12.9 | Yes | Yes | Yes | Hb | Cont. |
| DaSilva 2017 | USA | 55 | 6 | - | 55 | 73 | n/r | n/r | n/r | n/r | n/r | n/r | n/r | Yes | Yes | Yes | Hb | Dich. |
| English 2018 | Canada | 527 | 9 | - | 57 | 67.7 | n/r | 4 (med) | 40 | n/r | 52 | 8 | 19 | Yes | Yes | Yes | Hb | Cont. |
| Festic 2012 | USA | 318 | 9 | - | 54 | 64 | 2 (med) | 3 (med) | 39 | n/r | n/r | 8.5 | 22.6 | Yes | Yes | Yes | Hb | Cont. |
| Kim 2015 | South Korea | 211 | 9 | - | n/r | 63 | 35.1 | 79.6 | 17.5 | n/r | n/r | 9.5 | 35.5 | Yes | Yes | Yes | Hb | Cont. |
| Kramer 2008 | USA | 245 | 9 | - | 53 | 69 | 27 | 61 | 61 | n/r | 38.8 | 9.5 | 34.7 | Yes | Yes | Yes | Hb | Dich. & cont. |
| Kramer 2009 | USA | 245 | 7 | - | 53 | 69 | 27 | 62 | 61 | n/r | n/r | n/r | 35.1 | Yes | Yes | Yes | Hb | Cont. |
| Kumar 2014 | USA | 205 | 8 | - | n/r | 69.3 | 22.4 | 77.1 | 18 | n/r | n/r | 10 | 42 | Yes | No | Yes | Hb | cont. |
| Kumar 2017 | USA | 421 | 6 | clipping | 51.5 | 71 | 36.1 | 46 | 100 | n/r | n/r | 10 | 62 | Yes | Yes | Yes | Hb | cont. |
| Loustarinen 2015 | Finland | 488 | 3 | clipping | 56 | 60.9 | 40.6 | 81.6 | 100 | n/r | n/r | n/r | 11.1 (perioperative) | Yes | No | No | Hb | cont. |
| Naidech 2006 | USA | 103 | 6 | - | 55.3 | 63 | 29 | 54 | 60 | n/r | n/r | 10 | 46.6 | Yes | Yes | Yes | Hb | Cont. |
| Naidech 2007 | USA | 611 | 9 | - | 53.5 | 68 | 27 | n/r | n/r | n/r | n/r | 8 | 35.4 | Yes | Yes | Yes | Hb | Cont. |
| Pegoli 2015 | USA | 373 | 6 | - | 55.8 | 64 | 23.3 | 3 (med) | 35.7 | 35.7 | n/r | n/r | 23.9 | No | Yes | Yes | Hb | Cont. |
| Qi 2021 | China | 49 | 4 | elderly & H&H III-V | 72+ | 69.4 | 71.4 | n/r | 26.5 | 38.8 | 53.1 | n/r | n/r | No | Yes | No | 0 | Dich. |
| Sadamasa 2011 | Japan | 140 | 5 | aSAH + naSAH | 64 | 67.9 | 47.9 | 77.1 | 60 | 13.6 | n/r | n/r | n/r | Yes | No | No | RBC, Hct, Hb | Cont. |
| Sampson 2010 | USA | 243 | 9 | - | n/r | 63% | 23 | 3 (med) | 51 | n/r | 46.9 | 7 | 19.3 | Yes | Yes | Yes | Hct (Hb indirect) | Dich. |
| Smith 2004 | USA | 441 | 8 | clipping | 51 | 63.9 | 36 | 41.5 | 100 | 49.2 | n/r | 7 | 61.2 | Yes | Yes | Yes | Hb | Dich. |
| Springer 2009 | USA | 232 | 7 | aSAH + naSAH | 52 | 71 | 13 | n/r | n/r | n/r | n/r | 9 | 34.1 | No | Yes | Yes | Hb | Dich. |
| Stein 2015 | Germany | 522 | 8 | aSAH + naSAH | n/r | 59.4 | n/r | n(r | 59.5 | 32 | n/r | 8 | 48.9 | No | Yes | Yes | Hb & Hct | Dich. & cont. |
| Sun 2015 | China | 218 | 6 | - | 50+ | 63.8 | 14.2 | 41.7 | 64.2 | n/r | 35.3 | n/r | n/r | No | Yes | Yes | Hb | Cat. |
| Wartenberg 2006 | USA | 576 | 6 | aSAH + naSAH | 52.3 | 68 | 27.4 | n/r | 60.4 | 14 | 35.8 | 8 | 35.80% | No | Yes | Yes | Hb | Dich. |
| Naidech 2010 | USA | 44 | 5 | WFNS=2-4; CT Scale=3-4 | 59.2 | 75 | 29.5 | 97.7 | 75 | n/r | 82.6 | n/r | 88.6 | No | Yes | Yes | Hb | Dich. |
| Seicean 2015 | USA | 268 | 4 | clipping | n/r | n/r | n/r | n/r | 100 | n/r | 41.4 | n/r | 17.5 | Yes | No | No | Hct | Dich. |
| Yee 2017 | USA | 137 | 3 | clipping | n/r | n/r | n/r | n/r | 100 | n/r | n/r | n/r | 16.8 | Yes | No | No | Hb | Cont. |
| Zhang 2019 | China | 535 | 5 | - | 54.5 | 60.7 | 12.1 | n/r | n/r | 30 | n/r | n/r | n/r | Yes | No | No | Hb | Cont. |
| Wang 2019 | China | 554 | 4 | - | 51.6 | 60.3 | 12.5 | 39 | n/r | n/r | n/r | n/r | n/r | Yes | No | No | Hb | Cont. |
| Zheng 2019 | China | 760 | 4 | - | n/r | n/r | n/r | n/r | n/r | n/r | n/r | n/r | n/r | Yes | No | No | Hb | Cont. |
| Kurtz 2010 | USA | 34 | 5 | Neuro-monit. study | 52 | 68 | 83 | n/r | n/r | 29 | 60 | 7 | n/r | No | Yes | Yes | Hb | Dich. & cont. |
| Oddo 2009 | USA | 20 | 5 | Neuro-monit. study | 51 | 65 | 70 | 95 | n/r | 40 | n/r | n/r | n/r | No | Yes | Yes | Hb | Dich. & cont. |
| Dhar 2017 | USA | 37 | 5 | Iinter-vent. study | 54 | 89 | 28 | 67 | 50 | 53 | 50 | n/r | n/r | No | Yes | Yes | Hb | Cont. |
| Levine 2010 | USA | 421 | 8 | clipping | 51.5 | 63.9 | 34.9 | n/r | 100 | n/r | n/r | 10 | 50.8 | Yes | Yes | Yes | Hb | Dich. & cont. |
| Tseng 2008 | UK | 160 | 5 | - | 55.4 | 62.5 | 30 | 88.1 | 62.5 | n/r | n/r | 8 | 19.4 | No | No | Yes | Hb, Hct | Cont. |
| Schmitt 2022 | Germany | 5008 | 7 | - | 57.9 | 61.7 | n/r | n/r | 36.1 | 13.4 | 28.3 | n/r | 29.9 | Yes | No | Yes | Hb | Dich. & cont. |
| Li 2022 (a) | China | 218 | 3 | Propensity score | 53.6 / 52.4 | 50 | 17.4 / 12.8 | 76.1 / 77.1 | 67 / 70.6 | 29.4 / 22.9 | 27.5 / 43.1 | n/r | n/r | No | Yes | No | Hb | Dich. |
| Li 2022 (b) | China | 658 | 5 | Propensity score | 54.3 / 55.3 | 61.1 / 62.9 | 17.6 / 19.5 | 78.7 / 79.3 | 50 | 31.3 / 20.1 | 42.2 / 17.6 | n/r | n/r | No | Yes | No | Hb | Dich. |
| Wang 2022 | China | 351 | 6 | - | 56 | 65.8 | 41.6 | n/r | 83.2 | n/r | n/r | n/r | 8 | Yes | Yes | No | Hb | Cont. |

Abbreviations: aSAH = aneurysmal SAH, naSAH= non-aneurysmal subarachnoid hemorrhage, CT-scale = Columbia CT grading scale, NOS= the Newcastle-Ottawa Score for the quality assessment, H&H = Hunt & Hess score, WFNS= World Federation of Neurosurgical Societies, DCI = delayed cerebral ischemia, VS= vasospasm, RBCT = red blood cell transfusion, n/r= not reported, med – median.

**Supplementary Table S6:** Association between patients’ / SAH characteristics and early (peri-interventional) anemia / the need for perioperative RBCT. The risk of developing early/peri-interventional anemia or a need for perioperative RBCT is set out against each individual parameter. Significant associations are marked in green for the analyses with the evidence level I-II, or in light green for lower evidence. Non-significant associations are marked orange or light orange for the analyses with the evidence level I-II or lower, respectively.

| **Parameter** | **Early/peri-interventional anemia** | | | | | **The need for perioperative RBCT** | | | | |
| --- | --- | --- | --- | --- | --- | --- | --- | --- | --- | --- |
|  | Risk (odds ratio/as specified)  incl. 95% confidence interval | Nr. Studies | GQ Studies | MA | MVA | Risk (odds ratio/as specified)  incl. 95% confidence interval | Nr. Studies | GQ Studies | MA | MVA |
| Age (as defined) | Mean: 52.7±9.34 vs 50.9±10.8 yrs,p=0.41; r=-0.332,p<0.0001 | 2 | 1 | - | - | Mean: 59.0±8.6 vs 55.2±8.8 yrs, p=0.264 | 1 | 0 | - | - |
| Sex (female) | 9.26 (1.23 - 69.64) | 1 | 1 | - | - | 2.60 (0.48 - 14.02) | 2 | 0 | - | - |
| BMI |  |  |  |  |  | Mean: 22.6±1.9 vs 22.8±3.0 kg/m2, p=0.859 | 1 | 0 | - | - |
| Smoking | 1.32 (0.55 - 3.17) | 1 | 1 | - | - |  |  |  |  |  |
| Arterial hypertension | 0.52 (0.22 - 1.23) | 1 | 1 | - | - | 1.16 (0.26 - 5.13) | 1 | 0 | - | - |
| IA location  (anterior circulation) |  |  |  |  |  | n.s. | 1 | 0 | - | - |
| IA size (as defined) | IA>5 mm: 1.30 (0.51 - 3.30) | 1 | 1 | - | - | Mean: 5.6±2.3 vs 5.9±2.6 mm, p=0.759; per mm increase: 1.07 (1.01 - 1.41) | 2 | 0 | - | - |
| Initial clinical condition | 1.24 (0.71 - 2.14) | 2 | 1 | + | - | 2.71 (1.58 - 4.66) | 4 | 0 | + | + |
| Initial radiographic severity | Fisher scale: r=-0.332, p<0.0001; Hijdra score: 18.2±6.1 vs 18.3±5.86,p=0.91; IVH: 0.34 (0.08 - 1.47); Graeb score: 2.7±2.3 vs 2.9±2.7,p=0.11 | 3 | 2 | - | - | Fisher scale: 4.59 (1.39 - 15.13); IVH: 2.06 (0.76 - 5.62); ICH: 1.20 (0.36 - 3.97) | 2 | 0 | + | + |
| Acute hydrocephalus | Mean: 12.3±1.9 vs 12.9±1.8 g/dL,p=0.002 | 1 | 0 | - | - |  |  |  |  |  |
| Acute seizures  (within first week) | per g/dL increase: 0.97 (0.94 - 0.99) | 2 | 0 | - | - |  |  |  |  |  |
| Admission hemoglobin  (as defined) |  |  |  |  |  | Mean: 12.4±9.8 vs 13.0±1.5 g/dL, p=0.333 | 1 | 0 | - | - |
| Treatment modality (clipping) | 1.06 (0.44 - 2.55) | 1 | 1 | - | - |  |  |  |  |  |
| Duration of surgery |  |  |  |  |  | Mean: 277.3±88.0 vs 176.9±57.6 min, p=0.014 | 1 | 0 | - | - |
| IOAR |  |  |  |  |  | 19.48 (3.46 - 109.60) | 2 | 0 | + | + |

Abbreviations: SAH= subarachnoid hemorrhage, RBCT=red blood cell transfusion, MVA = multivariate analysis, MA =meta-analysis, IA= intracranial aneurysm, IOAR= intraoperative aneurysm rupture, GC = good quality, BMI = body mass index

**Supplementary Table S7**: Detailed description of the limitations and bias during data synthesis and analysis

| **Limitation/bias** | **Affected studies** |
| --- | --- |
| Variations in definition and timing of follow up and poor outcome, as well as the used diagnostic modality for the diagnosis of cerebral ischemia and infarction | All studies |
| No SD for Hb values mentioned:   - An arbitrary SD of 1.3 was used. - SD estimated upon the IQR values | - Kramer et al. 2009, Naidech et al. 2006, Stein et al. 2015. - Castella et al. 2021, Festic et al. 2013 |
| Poor outcome at 3 months is based on other publication. ^53^ No data available on poor outcome at discharge, therefore estimated at 50%. | Naidech et al. 2007 |
|  |  |
| No data available on outcome at discharge. Estimation based on median and IQR values. | Bell et al. 2015 |
| Possibility of overlapping cohorts, however, results included in same meta-analysis. | Naidech et al. 2006, Naidech et al. 2007 |
| Infarction rate calculated from reported OR for RBCT and DCI rate in patients with RBCT and rate of CTs in all patients | Kramer et al. 2008 |
| Forrest plot for vasospasm and mean Hb includes 2 studies using angiographic vasospasm and 1 study with TCD vasospasm | Bell et al. 2015, Naidech et al. 2006, Stein et al. 2015 |
| Forrest plot for vasospasm and nadir Hb includes 1 study with angiographic vasospasm and 1 study with clinical vasospasm | Naidech et al. 2010, Sun et al. 2015 |
| Forrest plot for vasospasm and admission Hb includes 1 study with angiographic vasospasm and 1 study with symptomatic vasospasm | Bell et al. 2015, Kim et al. 2015 |
| Forrest plot for vasospasm and Hb<10 g/dL includes 2 potentially overlapping cohorts, 2 studies with angiographic vasospasm, 1 study with symptomatic vasospasm and 1 study with TCD vasospasm | English et al. 2018, Kim et al. 2015, Kramer et al. 2008, Kumar et al. 2014, Kumar et al. 2017, Sampson et al. 2010 |
| Forrest plot for hospital stay duration:   - Anemia rate was estimated at 50% - SD and values estimated from IQR-values - median values applied instead of mean | Castella et al. 2021  Castella et al. 2021, Sampson et al. 2010 |
| Forrest plot for infectious complications: Wide range of RBCT thresholds between 8.0 -10.0 g/dL were included. | Kim et al. 2015, Levine et al. 2010, Kramer et al. 2008, Stein et al. 2015 |
| Forrest plot for age impact on anemia: Fictive SD value of 10 years according to the SD values from other studies was used | Kramer et al. 2008 |
| Forrest plot for Fisher grade impact on anemia risk: Data with regard to original or modified scale were included | English et al. 2018, Kramer et al. 2008, Kramer et al. 2009, Sampson et al. 2010 |
| Forrest plot for age impact on RBCT risk:   - SD estimated from IQR values - Fictive SD of 10 years | Festic et al. 2013  Kramer et al. 2008 |
| Forrest plot for initial clinical grade on RBCT risk: H&H and WFNS- based studies analyzed together | Broessner et al. 2009, Festic et al. 2013, Kim et al. 2015, Kramer et al. 2008, Kramer et al. 2009, Kumar et al. 2014, Kumar et al. 2017, Naidech et al. 2006, Levine et al. 2010 |

Abbreviations: SD= standard deviation; Hb= Hemoglobin; IQR= interquartile range, OR= odds ratio; WFNS= World Federation of Neurosurgical Societies
